# Assessment of lesion insertion tool in pelvis PET/MR data with applications to attenuation correction method development

**DOI:** 10.1002/acm2.14507

**Published:** 2024-09-04

**Authors:** Yutaka Natsuaki, Andrew Leynes, Kristen Wangerin, Mahdjoub Hamdi, Abhejit Rajagopal, Paul E. Kinahan, Richard Laforest, Peder E. Z. Larson, Thomas A. Hope, Sara St. James

**Affiliations:** ^1^ Keck School of Medicine of USC, University of Southern California Los Angeles California USA; ^2^ iSono Health Inc. South San Francisco California USA; ^3^ GE Healthcare Waukesha Wisconsin USA; ^4^ Washington University School of Medicine in St. Louis St. Louis Missouri USA; ^5^ University of California, San Francisco San Francisco California USA; ^6^ University of Washington Seattle Washington USA; ^7^ University of Utah Salt Lake City Utah USA

**Keywords:** attenuation correction, lesion insertion tool, PET/CT, PET/MR, PET reconstruction

## Abstract

**Background:**

In modern positron emission tomography (PET) with multi‐modality imaging (e.g., PET/CT and PET/MR), the attenuation correction (AC) is the single largest correction factor for image reconstruction. One way to assess AC methods and other reconstruction parameters is to utilize software‐based simulation tools, such as a lesion insertion tool. Extensive validation of these simulation tools is required to ensure results of the study are clinically meaningful.

**Purpose:**

To evaluate different PET AC methods using a synthetic lesion insertion tool that simulates lesions in a patient cohort that has both PET/MR and PET/CT images. To further demonstrate how lesion insertion tool may be used to extend knowledge of PET reconstruction parameters, including but not limited to AC.

**Methods:**

Lesion quantitation is compared using conventional Dixon‐based MR‐based AC (MRAC) to that of using CT‐based AC (CTAC, a “ground truth”). First, the pre‐existing lesions were simulated in a similar environment; a total of 71 lesions were identified in 18 pelvic PET/MR patient images acquired with a time‐of‐flight simultaneous PET/MR scanner, and matched lesions were inserted contralaterally on the same axial slice. Second, synthetic lesions were inserted into four anatomic target locations in a cohort of four patients who didn't have any observed clinical lesions in the pelvis.

**Results:**

The matched lesion insertions resulted in unity between the lesion error ratios (mean SUVs), demonstrating that the inserted lesions successfully simulated the original lesions. In the second study, the inserted lesions had distinct characteristics by target locations and demonstrated negative max‐SUV%diff trends for bone‐dominant sites across the patient cohort.

**Conclusions:**

The current work demonstrates that the applied lesion insertion tool can simulate uptake in pelvic lesions and their expected SUV values, and that the lesion insertion tool can be extended to evaluate further PET reconstruction corrections and algorithms and their impact on quantitation accuracy and precision.

## 1 INTRODUCTION

Positron emission tomography (PET) is a modality of choice for molecular imaging in oncology^1^, ^2^, ^3^ due to its sensitivity. The many imaging agents available today labeled with positron‐emitting radionuclides can inform about a variety of physiological markers such as metabolism, cellular proliferation, receptor status, and so on. Among the wide array of radiotracers, two prominent molecular probes such as ^18^F‐FDG and ^68^Ga‐PSMA inform about increased lesion glucose metabolism or prostate‐specific membrane antigen (PSMA) receptor status, respectively.^4^ In PET imaging, measured voxel intensity reflects radiotracer concentration in kBq/mL and thus enables diagnosis, staging, and quantitative assessment of disease in oncology. The standardized uptake value (SUV, unitless) is widely employed in clinical practice^5^ to compare uptake across patients, accounted for, and normalized with patient size (body weight) and radiotracer dose as well as imaging time relative to injection.

Imaging systems have become available that combine PET and magnetic resonance imaging (MRI), expanding beyond the ubiquitous use of PET/CT, to take advantage of the superior soft‐tissue contrast capabilities inherent to MRI. In 2011, PET/MR clinical scanners became commercially available.^3^, ^6^ As in PET/CT systems, the majority of PET/MR clinical applications is in oncologic imaging.^6^, ^7^, ^8^ With higher soft tissue contrast and multi‐parametric quantitative features of MRI as an advantage over CT, PET/MR provides added value in regions such as head and heck, liver, prostate, and pelvic bone metastases.^8^ The combination of newly developed radiotracers that are attached to novel pharmaceuticals and therapy targets^9^ with applicable MR sequences,^8^ staging, diagnosis and monitoring of disease progression may be improved with PET/MR.

While there continue to be ongoing advances in PET technology and algorithms, the attenuation correction (AC) calculated from matched CT images remains the single largest correction in PET image reconstruction.^10^ For the PET images to be quantitatively reliable and accurate, AC is essential to account for the loss of 511 KeV photons due to scattering and absorption. Any inaccuracy in AC hampers accurate measurement of lesion activity, which has clinical consequences in the longitudinal evaluation of cancer progression. In the context of clinical trials, accurate reproducibility is of paramount importance for repeatability, consistency, and reproducibility across sites.

For PET/CT systems, AC coefficients are obtained from a CT scan (i.e., CT‐based attenuation correction [CTAC]), where Hounsfield Units (HU) are converted to AC factors for 511 keV photons through linear scaling.^3^ These CT images are acquired in seconds and are used for anatomical reference as well.^11^ Anything that improves these CT images (e.g., CT truncation and metal artifacts corrections, iterative reconstructions, patient population, size‐dependent protocol parameters, etc.) affects the AC and thus the result of the PET reconstructions. On the other hand, PET/MR systems must rely on the nuclear spin properties of MR images for the synthetic CT and the AC (i.e., MR‐based attenuation correction [MRAC]). Obtaining accurate measurements of tissue densities from MRAC is particularly challenging for bones, which have a very short T2* relaxation time (T2 ∼ 0.4 ms^12^) that cannot be detected by conventional MR sequences. Initial implementations of AC algorithms from MR images were based on dual‐echo (2 point) Dixon MR sequence using two early echo times providing in‐phase (Water+Fat) and out‐of‐phase (Water−Fat) images exploiting the chemical shift of protons in fat relative to protons in water.^13^ These images were then used to derive water‐ and fat‐like images that were segmented as either water or fat‐like tissues with an assigned fixed known tissue attenuation coefficient.^14^ Inclusion of bones in the attenuation map in commercially available PET/MR systems has been addressed by incorporating the site‐specific bone atlas (e.g., pattern recognition algorithm akin to machine learning) into the multi‐parametric MR images.^15^, ^16^ While atlas‐based methods have shown improved accuracy and robustness, they rely upon average atlas bone structure and cannot address inter‐patient variability.^10^ Other MRAC methods provide identification of the bone structures by employing ultra‐short TE (UTE) MR sequences to capture the rapidly evanescent T2 signal of protons in bones.^15^ These direct imaging‐based methods address the patient variability issue. However, their AC accuracy is largely determined by the quality of the ensuing segmentation and CT conversion (e.g., linear HU vs. assigned HU for the segments).^10^ Multi‐parametric MRI with machine learning/deep learning approaches, a hybrid approach between atlas‐based (prior knowledge with training data) and direct imaging based (the MR‐based synthetic CT), are also currently under investigation.^16^

Evaluating the accuracy of these AC methods in patients is not trivial. Anthropomorphic phantom surrogates that meet the requirements for both PET (attenuation of the annihilation photons, electron density dependent) and MRI (magnetization of the atomic nuclei) are difficult to design.^17^, ^18^ Quantitative phantoms designed for MRI sequence optimization cannot be translated to PET (e.g., NIST quantitative phantoms for T1, T2, and diffusion MR sequences, which cannot be detected in PET scanners).

One viable option for evaluating image correction and reconstruction methods, such as the AC methods, is to utilize software‐based simulation tools. A lesion insertion tool is such software‐based simulation tools that allow physicians and physicists to simulate known volumes of uptake and evaluate image reconstruction methods, aiming to improve the clinical task of lesion detection and disease staging.^19^ Such tools allow for more realistic simulations, where accurate patient anatomy and tracer uptake can be modeled. Extensive validation of simulation tools is required to ensure that the simulated portions of a particular study match observed clinical features and, therefore, that results of the study are clinically meaningful. In a previous study, a lesion insertion tool was developed for the Siemens PET/MRI scanner (Siemens Healthineers, Knoxville, Tennessee) and validated using a phantom and a patient dataset.^20^ The previous study also harmonized the lesion insertion tool across the vendor; the lesion insertion tool they developed for Siemens Biograph mMR with the existing lesion insertion tool designed for the GE‐SIGNA PET/MR (General Electrics HealthCare, Waukesha, WI).^21^ The harmonization project was a crucial step in enabling the multi‐center clinical trial of novel PET tracers. The tool can be utilized to standardize the PET system reconstruction (vendor and system agnostic). This lesion insertion tool was integrated as part of the pipeline for the automatic evaluation of MRAC for neurological studies.^22^

In the current study, the lesion insertion tool developed for the GE PET scanners^23^ was validated in a clinical patient population. Lesions were inserted for both PET/CT and PET/MR studies with the goal to characterize differences in attenuation correction methods (CTAC vs. MRAC) and determine if these differences were consistent between the synthetic lesions and the measured lesions. We then extended the application of the lesion insertion tool to a second distinct cohort of patients, where lesions were inserted in four anatomical target locations within the pelvis with the goal of demonstrating the utility of such a tool to evaluate reconstruction parameters (including different ACs). The use of known synthetic lesions in clinical datasets enables direct evaluation of image correction and reconstruction methods on quantitation accuracy, including new MRAC methods.

## 2 METHODS

A previously described lesion insertion tool was employed and evaluated in a clinical patient data set for simultaneous PET/CT and PET/MR systems. The synthetic spherical lesions were validated with the matched lesion insertion. The lesions were matched in the reconstructed PET images, with the lesion size (diameter) and the reconstruction parameter (e.g., AC characteristics [CTAC vs. MRAC]). Taking full advantage of anatomical lateral symmetry in the pelvic region, inserting the synthetic spherical lesions contralaterally to the pre‐existing lesions will ensure the surrounding tissues are similar, and thus, the PET reconstructions can have similar back projection paths to the detectors (hereafter referred as the validation 1). Once the synthetic lesions were verified, the next step was to insert the synthetic lesions into the specific targeted regions without pre‐existing lesions in the patient cohort (*n* = 4) (hereafter referred as the validation 2).

### 2.1 Lesion insertion tool

The lesion insertion tool^23^ first inserts a synthetic spherical lesion in the image domain at a particular location with a given activity (voxel intensity, or concentration in Bq/mL, can be easily converted to SUVs). The inserted lesion image is then forward‐projected into a sinogram using the PET system model, which includes effects of Poisson noise, attenuation, scanner geometry, and normalization. The synthetic lesion sinogram is added to the original acquired patient sinogram. The combined sinogram is reconstructed into the final image, which is a hybrid image comprising the original patient with lesions and also the synthetic inserted lesion(s). In this process, the evaluated AC methods are incorporated into the PET system model, which is used in both the generation of the synthetic lesion sinogram and the reconstruction of the image. The benefit of employing a lesion insertion tool is that lesions of known activity can be inserted into known different regions of the anatomy (bone, soft tissue, etc.), and then the impact of different image correction and reconstruction methods can be evaluated.

In the current study, the use of different AC methods was tested and the impact on the resulting images and lesion volume of interest (VOI) SUVs was assessed. All the PET images in the study were reconstructed with time‐of‐flight (TOF) ordered subset expectation maximization (OSEM) algorithm, including a point‐spread function model,^24^ two iterations, and 16 subsets on an inplane field size of 600 mm, a matrix size of 256 × 256 × 89 and pixel size of 2.34 × 2.34 × 2:78 mm^3^ each.

### 2.2 Validation 1: Matched lesion insertion

**FIGURE 1:**
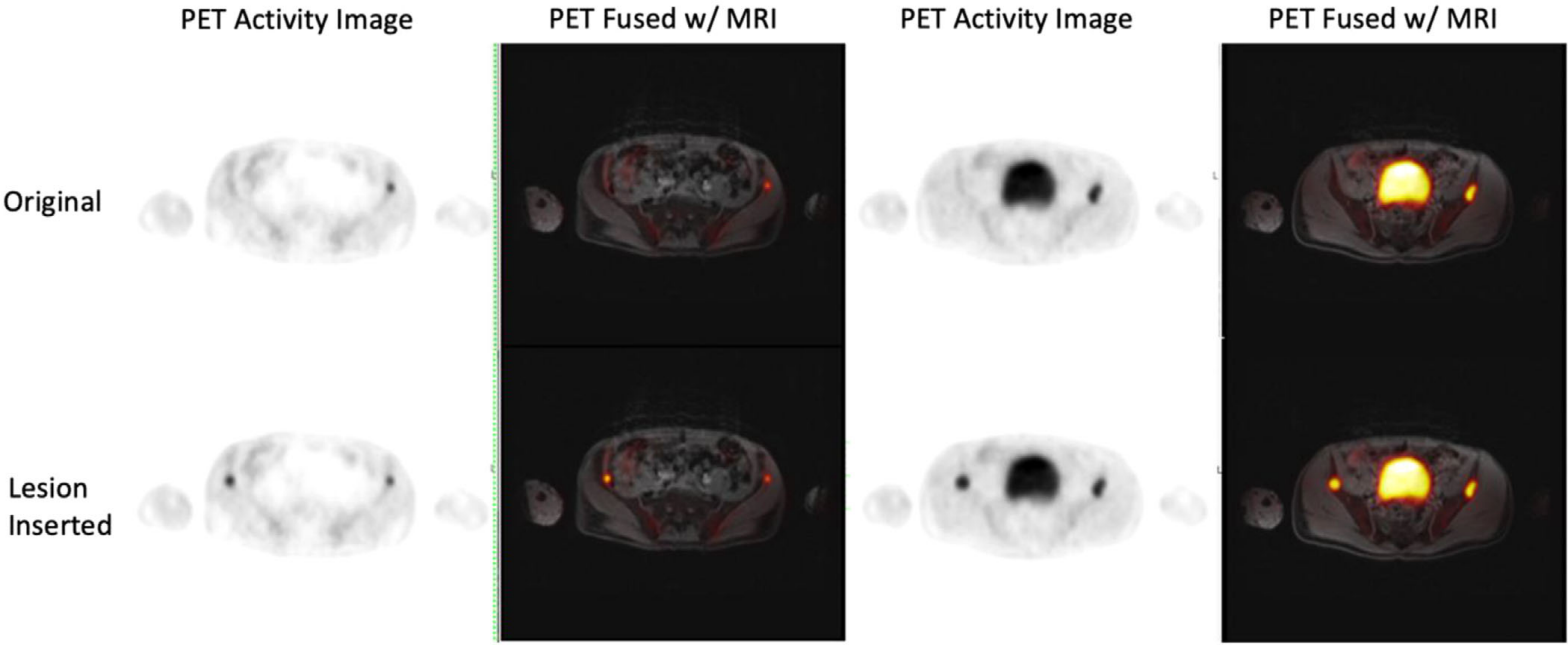
Representative pelvic patient PET/MR images showing original lesions (top row) with matched contralateral synthetic lesion insertions (bottom row). Two axial slices on the same patient PET/MR data are shown (odd columns are PET reconstructed images, even columns are PET images overlayed on corresponding MR water image slice).

In the patient cohort, a total of 71 lesions were identified in 18 pelvic PET/MR patient images acquired with a TOF simultaneous PET/MR scanner (SIGNA GE Healthcare, Waukesha, WI). For each patient's data, registered CTAC and MRAC (Dixon‐based AC without bones) were obtained. Synthetic lesions with matched contrast (mean SUV in the original lesion VOI) and size (matched spherical diameter) were inserted contra‐laterally and centered on the same axial slice (Figure 1). The CTAC and the MRAC were used for the reconstructions, and the differences in the calculated SUVs were evaluated for all lesions. For each original and synthetic lesion pair, the ratio of mean SUVs ([Mean MRAC]/[Mean CTAC]) was calculated, which is defined as the SUV Error Ratio. The matched pairs of the original and contra‐lateral lesions’ SUV Error Ratios were then divided (defined as the lesion error ratio) and analyzed across the lesion pairs (lesion error ratio vs. patient ID and vs. lesion diameter [range 9–40 mm]). In addition, concordance between the original and contralateral lesions’ SUV Error Ratio was evaluated with a Bland–Altman plot.

### 2.3 Validation 2: Targeted lesion insertion

**FIGURE 2:**
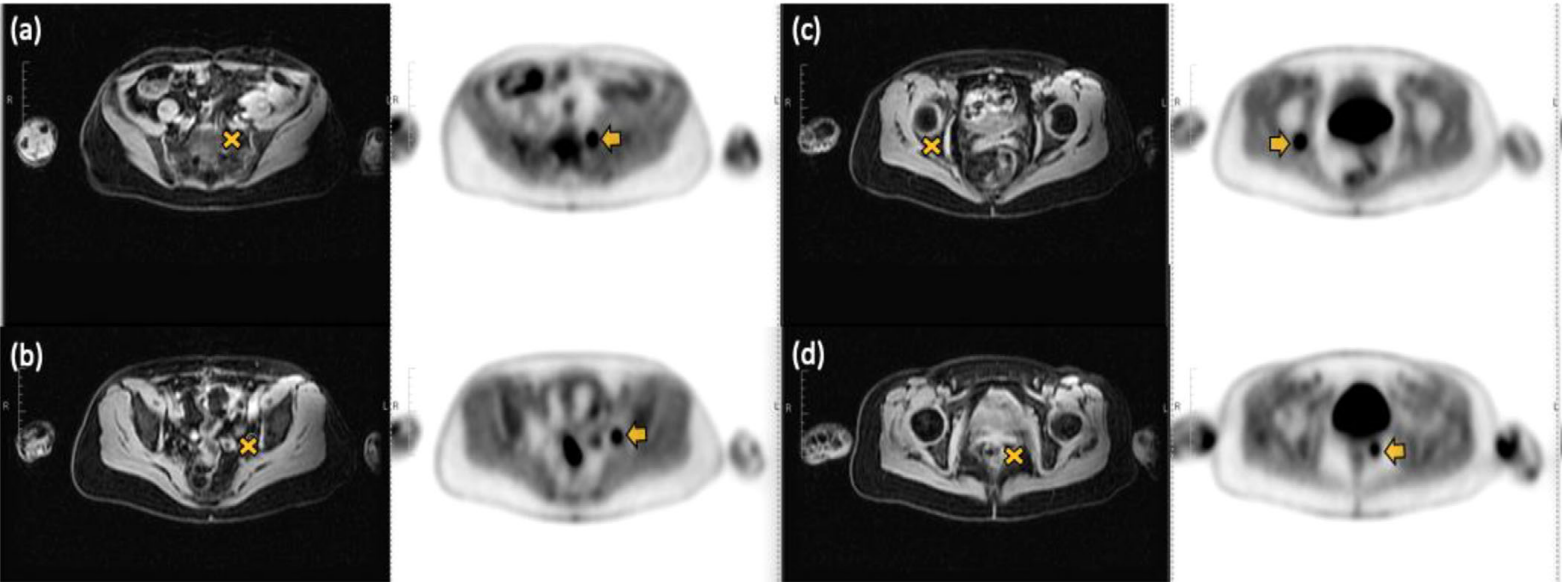
Representative pelvic patient PET/MR images with the synthetic localized lesion insertions. A total of four localized lesions are identified: (a) left mid sacrum, (b) pelvic sidewall lymph node, (c) right posterior acetabulum, and (d) rectum posterior bladder. For each lesion, structural MRI water contrast images (columns 1 and 3) are shown with the localized lesion sites (“X” is a visual representation of the central coordinates of the inserted spherical lesions) and the corresponding reconstructed PET images with synthetic inserted lesions (columns 2 and 4, arrows represent the inserted lesions).

The second portion of this work extends the application of the lesion insertion tool. Once the lesion insertion tool was validated in the patient cohort, four anatomical target locations with various compositions of soft tissues and bone (left mid sacrum, pelvic sidewall lymph node, right posterior acetabulum, and rectum posterior bladder) were identified in each of the four pelvic PET/MR patient studies acquired with the simultaneous PET/MR scanner (Figure 2). Every targeted lesion location was evaluated manually by a radiologist (TH) to ensure the absence of pre‐existing pathologic uptake. A total of 16 synthetic lesions with identical size (12 mm diameter) and contrast (activity concentration of 5000 kBq/mL) were inserted and reconstructed with both CTAC and MRAC.

For each synthetic lesion in the patient dataset, the distribution of the SUV_CTAC and SUV_MRAC within the targeted lesion VOI was evaluated. Then the difference percentage statistics were calculated as follows. First, the difference percentage for every voxel within the VOI was calculated using:

(1) $$\begin{array}{ccc} & & \mathrm{Differenc}e_{\mathrm{voxel}}\;\left(\%\right)=\;100*\\ & & \left(\frac{\mathrm{SUV}\_\mathrm{MRA}C_{\mathrm{voxel}}-\mathrm{SUV}\_\mathrm{CTA}C_{\mathrm{voxel}}}{\mathrm{SUV}\_\mathrm{CTA}C_{\mathrm{voxel}}}\right) \end{array}$$

where SUV_MRAC_voxel_ is the calculated SUV in a voxel using the MR AC method, and SUV_CTAC_voxel_ is the calculated SUV in a voxel when the CT‐based AC method is applied. Second, the maximum difference percentage (diff%max, the largest discrepancy in SUV_MRAC_voxel_ vs. the ground truth SUV_CTAC_voxel_) was identified within each VOI and reported in the box plot for each of the 4 localized lesion anatomical targets. The SUV error values, the $Difference_{voxel}\left(\%\right)$ across the lesion, were analyzed across the four patients’ data for mean (*μ*), standard deviation (*σ*), and root‐mean‐squared error (RMSE = $\sqrt{\mu^{2}+\sigma^{2}}$) and the maximum.

## 3 RESULTS

### 3.1 Derived from validation 1: Matched lesion insertion

**FIGURE 3:**
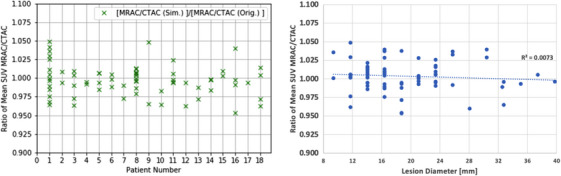
Evaluation of the lesion error ratios versus patient number (left) and as a function of lesion diameters (right).

**FIGURE 4:**
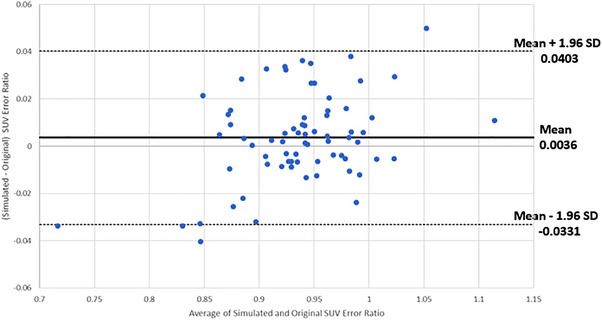
Bland–Altman plot of the SUV error ratios for contralateral simulated and original lesions.

The lesion error ratio was plotted versus patient number ID and lesion diameter (Figure 3). The Lesion Error Ratio over lesion pairs was unity (mean Lesion Error Ratio 1.004 ±0.020), which indicates that the synthetic and original lesions have similar SUV error characteristics from CTAC and MRAC. There were no linear trends when evaluating the Lesion Error Ratio versus lesion diameter size (*R* ^2^ = 0.0073). The Bland–Altman analysis (Figure 4) shows that the differences were within a 95% confidence interval, indicating there were no significant differences in the calculated SUV Error Ratios.

### 3.2 Derived from validation 2: Targeted lesion insertion

**FIGURE 5:**
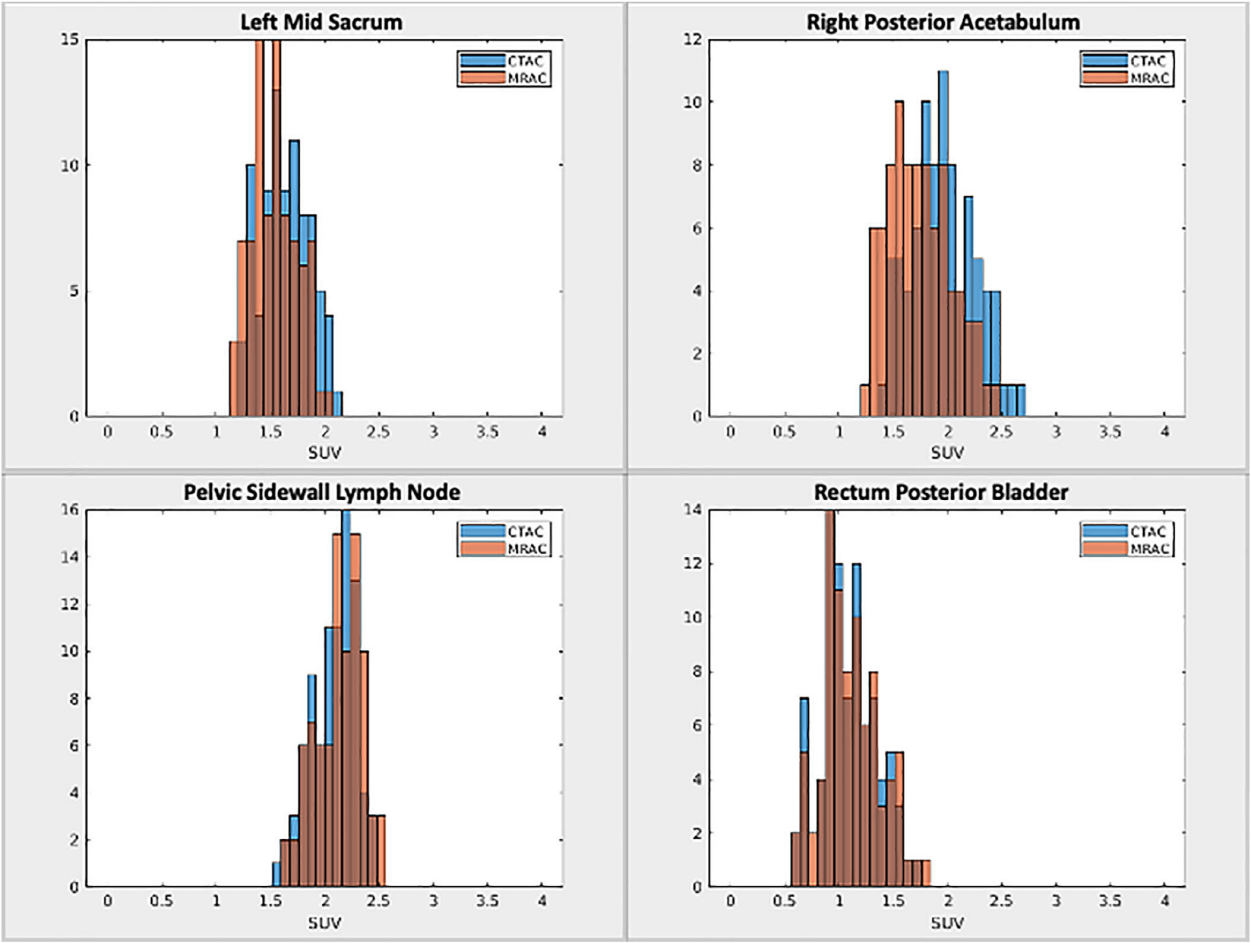
Representative histogram of voxels for SUV_CTAC (blue) and SUV_MRAC (orange) in the synthetic lesion VOIs.

**FIGURE 6:**
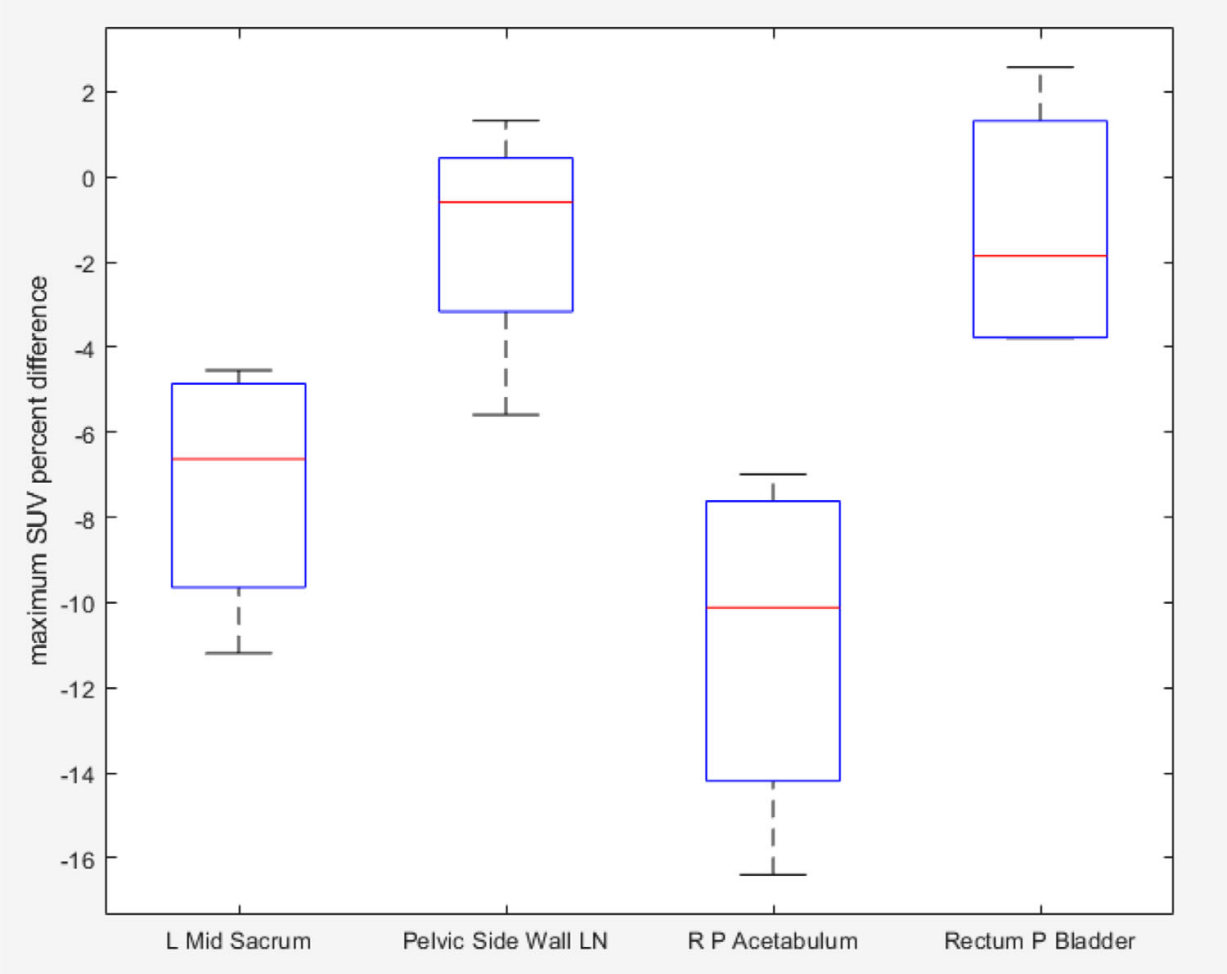
Box plots of maximum SUV percent difference for each of the anatomical target lesion locations across four patients.

The histograms of lesion SUVs in the four anatomical target locations show distinctive distribution patterns (Figure 5). When the VOI consisted of a higher proportion of bone than soft tissue and was located near bones, negative mean differences were observed. The box plots (Figure 6) summarize the calculated lesion's SUV across all patients for each anatomical target location. Of the four targets, the lesion in the right posterior acetabulum has the largest fraction of bone in the lesion VOI and has the largest percent difference in the negative direction (*μ* −10.91%, *σ* 4.23%, RMSE 11.70%), followed by the left mid sacrum (*μ* −7.25%, *σ* 3.05%, RMSE 7.87%). With these bone‐dominant structures, MRAC that does not take bone signals into account (which is the case with our Dixon‐based MRAC) is expected to have lower SUVs due to under‐correction of attenuation. The other two lesions (pelvic side wall lymph nodes [*μ* −1.36%, *σ* 2.96%, RMSE 3.26%] and rectum posterior bladder [*μ* −1.23%, *σ* 3.12%, RMSE 3.35%]) comprise predominantly soft tissues, resulting in lower SUV errors. The voxel‐wise difference for the soft tissue dominant lesions was much closer to zero, unlike the bone dominant lesions.

## 4 DISCUSSION

The lesion insertion tool that was employed in this work was originally developed for the purpose of evaluating and comparing lesion detectability in different reconstruction algorithms and methods.^19^, ^23^ The previous work on the tool evaluation was done with a whole‐body patient dataset but did not specifically validate the tool by using matched inserted lesions.^20^, ^23^ The current study is an extension to the previous tool evaluation work, as an extensive pelvic PET/MR patient data set is employed to simulate the real‐world clinical applications (e.g., simulating the realistic lesions, inserting synthetic lesions in any anatomical target locations, utilizing these as ground truth emission activities for the evaluation of the different reconstruction methods and algorithms, and enabling multi‐center clinical trials with standardized PET reconstruction methods).

The current study first assessed the validity of the lesion insertion tool in a patient cohort, by evaluating the quantitative accuracy of different PET/MR AC methods using contra‐laterally matched synthetic lesions with a PET/MR lesion insertion tool. The analysis is based on the hypothesis that contra‐laterally matched synthetic lesions within the same patient data will have similar bone and soft tissue compositions as within the original lesion VOI and will be surrounded by similar organs. Using this framework, the reconstructed images using CTAC and MRAC methods have similar AC errors and, therefore, similar SUV error ratios between the synthetic and the original lesions. The results presented herein demonstrate that the synthetic lesion reproduces the original lesion in the reconstruction process, and trends observed in SUV errors using CTAC and MRAC were consistent between the synthetic and original lesions.

Once the lesion insertion tool was validated in the first clinical data set, synthetic lesions were inserted into four anatomical target location regions in the pelvis in a cohort of patients that did not have any observed clinical lesions in the pelvis. Four target locations with distinctive voxel composition of predominantly soft tissue and bones, with the goal to illustrate how the lesion insertion tool can be applied to evaluate the impact of different AC methods on the quantitation of PET images. The results show that lesions within each anatomical target location demonstrate similar SUV quantitation error traits and percentage SUV difference statistics.

The current study has a few limitations. First, the application of the lesion insertion tool in this study is limited to pelvis data, which is an area of the body with large bones that can have an impact on AC and quantification. However, the impact of bone on quantitation observed in this work should be minimized in other anatomical sites, such as the head and neck. Second, the method of evaluating the equivalency of the contralateral synthetic lesions compared to the original lesions was solely based on a comparison of the SUV errors and differences using the most common MRAC and CTAC methods that have existing discrepancies and impact on resulting SUVs. While other promising MRAC methods that narrow the gap with the CTAC methods (the ‘ground truth’) are available, the purpose of the current study was to validate the lesion insertion tool and not to optimize the MRAC methods. The lesion SUV percent difference for the anatomical target location closest to bone was smaller (*μ*−7.25%) than what other groups have reported previously (∼ 10% or greater when close to or in bone^15^, ^25^). This difference could be explained by tissue composition in the VOIs; our analysis was based on in vivo pelvis patient data with high heterogeneity of tissues contained in spherical VOIs at each anatomical target location, while the other groups’ analyses were based on VOIs defined in a homogeneous phantom dataset of specific tissue types and/or better delineated tissue types. Lastly, this study used a PET scanner with TOF, which has been shown to reduce quantitation errors associated with incorrect AC values^25^, ^26^; a similar study using a non‐TOF PET scanner would have shown larger SUV differences for VOIs in soft tissue away from the bone, similar to the differences seen in VOIs in this study near the bones.

While the focus of our current work evaluated and compared AC methods, the lesion insertion tool can be applied to evaluate reconstruction algorithms and methods in a similar fashion. With PET's overall clinical goal of lesion detection and disease staging, image reconstruction methods remain as an active field of research to improve spatial resolution, decrease image noise, and enhance target‐to‐background contrast.^19^ The optimization of the tunable parameters (e.g., number of iterations, priors, filtering, etc.) can impact the reconstruction, and the lesion insertion tool can evaluate these with the reconstructed SUVs in the synthetic lesions as a metric. Similarly, novel reconstruction methods, such as advanced statistical methods (e.g., Bayesian penalized likelihood image reconstruction)^27^ and data‐driven methods (e.g., neural network noise reduction),^28^ can be evaluated with the lesion insertion tool; set up 2 sets of simulations with the user‐defined delta in reconstruction methods, and compare these two simulations with mean SUVs as a metric once again. The lesion insertion tool, however, is not designed to identify where in the reconstruction steps that caused the SUVs errors. A careful design of the two‐simulations study is paramount, ensuring only one aspect of the reconstruction is altered with clear intention (thus “user‐defined” delta) and keeping everything else the same.

## 5 CONCLUSION

The presented work demonstrates that the applied lesion insertion tool can simulate uptake in pelvic lesions and their expected SUV values, and that synthetic lesion insertion can be used to further evaluate PET reconstruction corrections and algorithms and their resulting impact on quantitation accuracy and precision. The current validation work further strengthens the confidence in using this tool and similar approaches for evaluating novel AC methods that have the potential to improve quantitative accuracy in simultaneous PET/MR systems. The lesion insertion tool is now primed for further studies with different PET reconstruction and correction methods, similar to this study focusing on AC methods.

## AUTHOR CONTRIBUTSIONS

Yutaka Natsuaki: Expertise in medical imaging and MR, conception and design of the study, technical development of the software/tools, data analysis, interpretation of results, draft and revision of the manuscript, and final approval of the version to be submitted. Andrew Leynes: Expertise in lesion insertion tool and PET/MR and PET/CT, technical development of software/tools, data analysis, interpretation of results, and revision of the manuscript. Kristen Wangerin: Expertise in lesion insertion tool (GE) and PET/MR and PET/CT, data analysis, interpretation of results, critical revision of the manuscript (especially with the intellectual contents), and final approval of the version to be submitted. Mahdjoub Hamdi and Abheijit Rajagopal: Data analysis, interpretation of results, and revision of the manuscript. Paul E. Kinahan and Richard Laforest: Expertise in PET/MR and PET/CT, data analysis, interpretation of results, revision of the manuscript, and final approval of the version to be submitted. Peder E.Z. Larson: Expertise in PET/MR and PET/CT, data collection, facilitating data analysis resources, data analysis, interpretation of results, revision of the manuscript, and final approval of the version to be submitted. Thomas A. Hope: Expertise in PET/MR and PET/CT, clinical data collection, facilitating data analysis resources, contribution in clinical analysis (e.g., identifying four targeted regions in Validation 2), interpretation of the results, revision of the manuscript, and final approval of the version to be submitted. Sara St. James: Expertise in PET/MR and PET/CT, conception and design of the study, data analysis, interpretation of results, critical revision of the manuscript, and final approval of the version to be submitted.

## CONFLICT OF INTEREST STATEMENT

Wangerin, Kirsten is an employee of General Electrics Healthcare. The other authors have no conflicts of interest to disclose.
